# Understanding and communicating epidemiological measures of risk and benefit

**DOI:** 10.1093/fampra/cmac117

**Published:** 2022-10-18

**Authors:** Caroline de Moel-Mandel

**Affiliations:** Department of Public Health, School of Psychology and Public Health, La Trobe University, Melbourne, Australia

## Introduction

Practically, every intervention decision that is made in the primary health care setting requires the application of a health benefit versus risk assessment. While there are many expert guidelines available that can help health practitioners with the decision-making process of, for example, cardiovascular disease and diabetes management, it is important that the supporting data used to develop these guidelines is understood as well. The effectiveness and safety of an intervention, preferably evaluated in a randomized clinical trial, is generally expressed in relative terms, such as the epidemiological measure “relative risk.” Absolute measures of risk, which predict the intervention’s benefit for an individual patient, are not always assessed or included in guidelines and therefore not commonly used by health practitioners to develop appropriate health care plans.^1^

It is recognized that most people do not have the required health care literacy and numeracy skills to understand health risks discussions in scientific research and to use this information to make fully informed decisions about their medical care or treatment.^2^ It is therefore the task of health practitioners to effectively and correctly communicate benefits and risks of proposed interventions with their patients, preferably with the help of decision aids such as brochures, videos, or web-based tools.^3^ Consequently, a health practitioner struggling to understand basic statistical concepts might miscommunicate risk, which may affect a patient’s ability to provide informed consent.^2^ This could ultimately result in patients making a decision that is incongruent with their preferences and values.^1^

This article offers a deeper understanding of some of the epidemiological measures used to report the safety and effectiveness of health interventions. The following explains what relative and the absolute risk measures are, and how they can be calculated, interpretated and applied. Further insight is provided with the help of 3 relevant examples.

## Epidemiological measures to report effectiveness and safety of health interventions

The effectiveness and safety of health interventions needs to be demonstrated with the epidemiological measures relative risk reduction (RRR) and absolute risk reduction (ARR) (also called attributable risk). Both measures provide a different sense of the clinical importance of an intervention. The RRR reflects the percentage reduction in disease for those undergoing the intervention compared with those not undergoing the intervention. The ARR is a measure of risk difference and reflects the excess risk of disease associated with not receiving the intervention. The terms are often misunderstood, misperceived, and erroneously directly compared.^4^ The easiest way to illustrate their difference is with the use of a hypothetical clinical trial:

Imagine a study in which 200 people are randomly allocated to either a group receiving an intervention or to a control group receiving a placebo intervention. All participants are followed over a specified time and regularly checked for the occurrence of a disease. At the end of this period, 12 people in the intervention group and 20 in the placebo group developed the disease of interest. The risk (or incidence) of disease can be calculated for each group by dividing the number of people who acquired the disease over the total number of participants in the group. The hypothetical study findings of this example indicate that the risk of disease in the placebo group (the baseline risk) is 20 in 100 participants (20%) and the risk of disease in the intervention group is 12 in 100 participants (12%) (see Table 1).

**Table 1.** A 2 × 2 contingency table for disease in a hypothetical intervention trial.

Intervention	Disease			
		Yes	No	Total
Yes	12	88	100
No	20	80	100
Total	32	168	200

$\begin{array}{l} \begin{array}{ll} & Risk\;of\;disease\;in\;intervention\;group\;=\frac{12}{100}\;=\;12\%;\\ & Risk\;of\;disease\;in\;control\;group\;=\frac{20}{100}=20\%. \end{array} \end{array}$

**Fig. 1.**
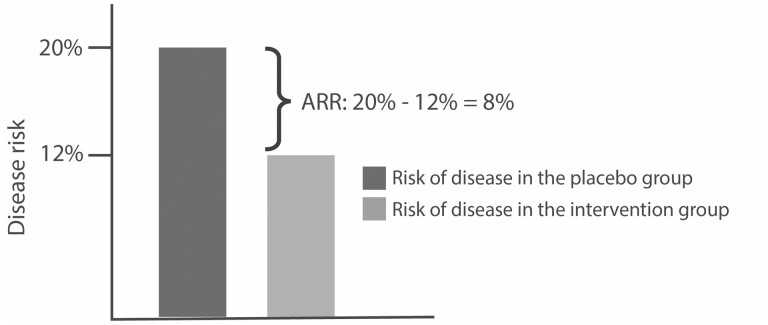
The absolute risk reduction in a hypothetical study with a disease risk in the placebo group of 20% and a disease risk in the intervention group of 12%.

The relative risk (RR) of developing the disease is expressed as the ratio of the risk in the intervention group to that in the placebo group, or $\frac{12\%}{20\%}\;=\;0.6$. An RR with a value lower than one implies that there is a lower risk of disease in the intervention group, which suggests that the intervention might be effective. To understand the real value of the clinical intervention, the results are subsequently reported as a RRR and ARR.^5^ The RRR is the amount by which the intervention has reduced the RR. It is calculated from the RR and expressed as a percentage as follows: RRR = (1 − RR) × 100. The RRR in this study is therefore (1 − 0.6) × 10 = 40%. In other words, the intervention has reduced the risk for disease by 40%. The ARR is defined as the absolute difference between the risk of disease in the placebo group and the risk of disease in the intervention group: ARR = Risk in placebo group − Risk in intervention group.^5^ In the example above, the ARR is 20% − 12% = 8%, indicating that the intervention has prevented the disease in 8% of the patients. Simply put, there are 8 fewer cases of disease per 100 people who received the intervention (see Fig. 1). Another way of communicating this is with the number needed to treat (NNT), calculated as 1/ARR. In this example, the NTT is 1/0.08 (8%) = 12.5, which implies that approximately 13 patients need to receive the intervention to prevent one new case of the disease. This indicates that the intervention is quite effective.

While the RRR is commonly used in papers to provide information about the effectiveness of an intervention, the measure does not consider the actual risk of disease in a population. For example, a study might report about a new intervention with an impressive 50% disease reduction. In a population where the risk of the disease is only 0.004%, the new intervention will reduce the disease risk to 0.002%, an improvement that is not actually of any clinically relevance.^6^ The baseline disease risk in a population, however, does have an influence on the ARR. To demonstrate this, we will decrease the baseline risk of disease in the placebo group in the above hypothetical study from 20 in 100 people (20%) to 2 in 100 people (2%). Because we will keep the ratio of the risks the same (RR = 0.6), the risk of disease in the intervention group will now become 1.2 in 100 people (1.2%). Those percentages result in an ARR of 0.8% (2% − 1.2%), which indicates a much lower benefit of the intervention for the population as now there will be only 0.8 fewer cases of disease per 100 people who received the intervention. By contrast, if the disease is very prevalent in the population and the baseline risk of disease in the placebo group becomes 40%, then the benefit of the intervention will increase substantially. The ARR of 16% (40% − 24%) indicates that there will be 16 fewer cases of disease for every 100 people who receive the intervention (see Table 2).

**Table 2.** The relationship between baseline disease risk and absolute decrease in risk (absolute risk reduction), with relative risk = 0.6.

Baseline risk of disease in control group (%)	Risk of disease in intervention group (%)	RRR (%)	ARR (%)
2	1.2	40	0.8
20	12	40	8
40	24	40	16

In conclusion, when only RRs or RRRs are reported, the clinical significance of the health intervention cannot be established. Including absolute numbers of additional cases will provide a much better picture of the overall situation.^7^

## The RRR and ARR in context

The difference between and the relevance of the 2 measures on a population and individual level is further demonstrated with the following randomly chosen examples.

A systematic review by Chapelle et al.^8^ suggested high consumption of fruits and vegetables to be associated with a significant reduced risk of 49% in colorectal cancer (RR = 0.51). No absolute risk statistics were reported. The article was criticized by Lawrence et al.,^9^ who, with the provided information and a 2% baseline risk of colorectal cancer in the global population, estimated the ARR to be quite small, fewer than 1 in 100 people (1%). For the individual patient, this diet might not seem very worthwhile. On a global population level on the other hand, a high consumption of fruits and vegetables can potentially prevent approximately 680,000 cases of colorectal cancer and 350,000 global deaths each year.^9,10^

Another example relates to the early side effects of COVID-19 vaccines. Surveillance during the initial 6 months of vaccine distribution showed a high RR of 3.75 for myocarditis/pericarditis following the first 3 weeks after COVID-19 vaccination among individuals aged 12–39 years.^11^ While the almost 4-fold increased risk appeared alarming, the absolute risk for disease turned out to be very low, corresponding with only 6 extra cases per million administered vaccine doses.^11^ Considering the global spread of COVID-19 infections and the potential long-term after effects of the disease among this age group, the vaccine’s overall benefits far outweigh the low absolute risk on the individual as well as a global level.^12^

The first COVID-19 vaccine trials generally reported effectiveness solely with RRR measures. Those measures ranged from 67% for the AstraZeneca–Oxford vaccine to 95% for the Pfizer–BioNTech.^13^ At that time, the much lower AstraZeneca–Oxford RRR might have given the impression that this vaccine was less effective. However, effectiveness evaluations need to include the ARRs as well as the trial circumstances.^14^ Due to the higher baseline infection risk throughout the AstraZeneca–Oxford trial, the ARR for this vaccine (1.3%) was higher than the Pfizer–BioNTech ARR (0.84%).^14^ Furthermore, the vaccine trials were performed under strictly controlled but different conditions and with healthy individuals. Reported benefits will therefore prove to be less applicable to a broader population that includes people of all ages with varying health challenges and residing in different health systems.^15^

## Conclusion

The effectivity and safety of health interventions are generally reported as RR measures. High-risk percentages might indicate that a treatment, screening, test, or vaccination has a drastic impact on the disease or health state in the population. However, in the health care setting, where the focus is on the benefit for the individual patient, RRs must be evaluated in close relation to absolute risk measures to account for the baseline risk of disease. Health practitioners need to understand both statistical concepts to assess the clinical significance of health interventions and to apply clinical evidence to their practice. By correctly communicating risk, with the help of available decision aids, patients will be able to make informed decisions about the consequences of interventions proposed for themselves and their family.
