# Correction to: Understanding and communicating epidemiological measures of risk and benefit

**DOI:** 10.1093/fampra/cmac152

**Published:** 2023-01-03

This is a correction to: Caroline de Moel-Mandel, Under­standing and communicating epidemiological measures of risk and benefit, Family Practice, 2022, cmac117, https://doi.org/10.1093/fampra/cmac117

In the originally published version of this manuscript, the following two lines belonging to Table 1 were erroneously placed in the Conclusion section:

$\begin{array}{l} \begin{array}{ll} & Risk\;of\;disease\;in\;intervention\;group\;=\frac{12}{100}\;=\;12\% \end{array} \end{array}$

$\begin{array}{ll} & Risk\;of\;disease\;in\;control\;group\;=\frac{20}{100}=20\% \end{array}$

In addition, the caption of Table 1 as well as row and column headings were incorrect.

These errors have been corrected.

